# Same day discharge after robot assisted partial nephrectomy

**DOI:** 10.1007/s00345-025-05968-9

**Published:** 2025-10-12

**Authors:** Ronny Baqain, Laith Baqain, Sohrab Ali, Simone Crivellaro, David Lee, Ryan W. Dobbs, Mohammad Shahait

**Affiliations:** 1 University of Manchester, Manchester, UK; 2 Division of Gastroenterology and Hepatology, New York-Presbyterian Weill Cornell Medical Center, New York, NY USA; 3 Department of Urology, University of California, Irvine, USA; 4 Department of Urology, Robotic Urology, UIC Urology at Mount Sinai Hospital, University of Illinois at Chicago, College of Medicine, University of Illinois Hospital Health Sciences System, Chicago, USA; 5 Department of Urology, Cook County Health & Hospitals System, Chicago, USA

**Keywords:** Ambulatory surgical procedures, Robotics, Minimally invasive surgical procedures, Partial nephrectomy, Nephron sparing surgery, Kidney tumors, Renal carcinoma

## Abstract

**Purpose:**

Partial nephrectomy (PN) is the standard treatment for small renal masses, offering effective cancer control while preserving renal function. The adoption of robotic assisted partial nephrectomy (RAPN) has improved perioperative outcomes, and there is a growing interest in same-day discharge as a strategy to enhance recovery, reduce healthcare costs, and achieve more favourable postoperative outcomes. This review evaluates the current evidence surrounding SDD following RAPN, focusing on perioperative outcomes, patient selection, and evolving surgical practices.

**Methods:**

A comprehensive literature search identified seven studies reporting outcomes of SDD RAPN, including a total of 485 patients. The findings suggest that SDD can be implemented safely, with complication, readmission, and emergency department visits similar to those observed in inpatient cohorts. Enhanced Recovery After Surgery (ERAS) protocols and minimally invasive surgical techniques such as single-port and retroperitoneal approaches, play an important role in facilitating early discharge by reducing postoperative pain, enhancing recovery, and shortening hospital stays.

**Results:**

Effective implementation of SDD relies on appropriate patient selection, surgeon experience, and institutional infrastructure. Future directions include refining discharge criteria, standardizing ERAS protocols, and incorporating postoperative tools such as telemedicine and wearable technologies.

**Conclusion:**

SDD after RAPN is both feasible and safe in selected patients. Broader adoption will depend on the development of standardized pathways, robust patient selection strategies, and additional research to validate outcomes across clinical settings.

## Introduction

 Partial nephrectomy (PN) is the standard treatment for small renal masses, offering effective cancer control while preserving kidney function and reducing risk of chronic kidney disease [1–3]. Minimally invasive techniques, especially robot-assisted partial nephrectomy (RAPN), have gained popularity due to improved perioperative outcomes and faster recovery [4]. RAPN has also helped extend the indications for partial nephrectomy, allowing surgeons to safely treat more challenging cases that might have previously required radical nephrectomy [5]. Its adoption has steadily increased, coinciding with a decline in open and purely laparoscopic approaches [6], driven by technological advances, improved surgeon expertise, and greater access to robotic platforms in various clinical settings [7].

The Covid-19 pandemic placed an unprecedented strain on healthcare systems around the world, challenging hospitals to optimize resources and reassess perioperative pathways. In this context, same-day discharge protocols, particularly following procedures like RAPN, gained momentum as a way to reduce inpatient stays, limit exposure risk, and conserve capacity [8, 9]. This trend was also observed in other urologic procedures such as robotic-assisted prostatectomy, where same-day discharge protocols demonstrated safety and efficiency in carefully selected patients [10].

Recent advancements in robotic surgery have contributed significantly to the growing feasibility of same-day discharge (SDD) after RAPN. One of the most notable innovations is the introduction of single-port (SP) robotic platforms, which utilize a single incision to access the surgical field [11, 12]. This approach minimizes tissue trauma, reduces postoperative pain, and often allows for faster recovery compared to traditional multi-port (MP) systems [13, 14]. Additionally, the retroperitoneal approach offers another minimally invasive route bypassing potentially challenging transperitoneal operations by avoiding manipulation of intraperitoneal organs, leading to reduced gastrointestinal disturbance, less blood loss, and a shorter length of stay [15]. Enhanced Recovery After Surgery (ERAS) protocols have further supported these efforts by standardizing perioperative care to optimize and facilitate early discharge [16]. Together, these techniques are enabling a shift toward more streamlined postoperative pathways, with increasing reports of SDD being safely implemented in centers of excellence. As the technology continues to evolve and surgical teams gain more experience, these methods are likely to play an even larger role in promoting early discharge without compromising patient safety or outcomes [13].In this context, we aimed to review the current evidence that support the feasibility and safety of SDD after RAPN. Also, we sought to propose a framework to guide future perioperative management and research directions.

## Methods

For this review, we included studies that reported patient outcomes following RAPN and involved at least some patients who were discharged on the same day as their surgery. Following the PICO framework, the review’s scope was defined as: P- Patients with kidney tumours; I- those who underwent RAPN with same-day discharge (outpatient surgery); C- Compared to those who had inpatient surgery; O- Perioperative outcomes of interest.

### Search strategy

In March-April 2025, we conducted a comprehensive search of medical databases to identify studies evaluating outcomes of SDD following RAPN. The databases searched included PubMed/Medline, Science direct, and Google Scholar. The search terms used were “partial nephrectomy OR nephron sparing surgery OR robotic assisted partial nephrectomy OR minimally invasive partial nephrectomy AND same-day discharge OR ambulatory OR day case OR outpatient.” SDD after RAPN was defined as discharge of patients on postoperative day 0.

### Inclusion/Exclusion criteria

Studies were included if they met the following criteria: (1) Patients underwent RAPN; (2) Outcomes were specifically reported for a SDD cohort; (3) The study was published between 2000 and 2025; and (4) The study reported at least one of the following: complication or readmission rates, operative details, healthcare costs, quality of life, patient satisfaction, information on SDD/ Enhanced Recovery After Surgery (ERAS).

Studies were excluded if (1) Outcomes were not distinguished between SDD and inpatient groups, or (2) the study design was a case report, review article, expert opinion, or commentary.

### Data extraction

**Fig. 1:**
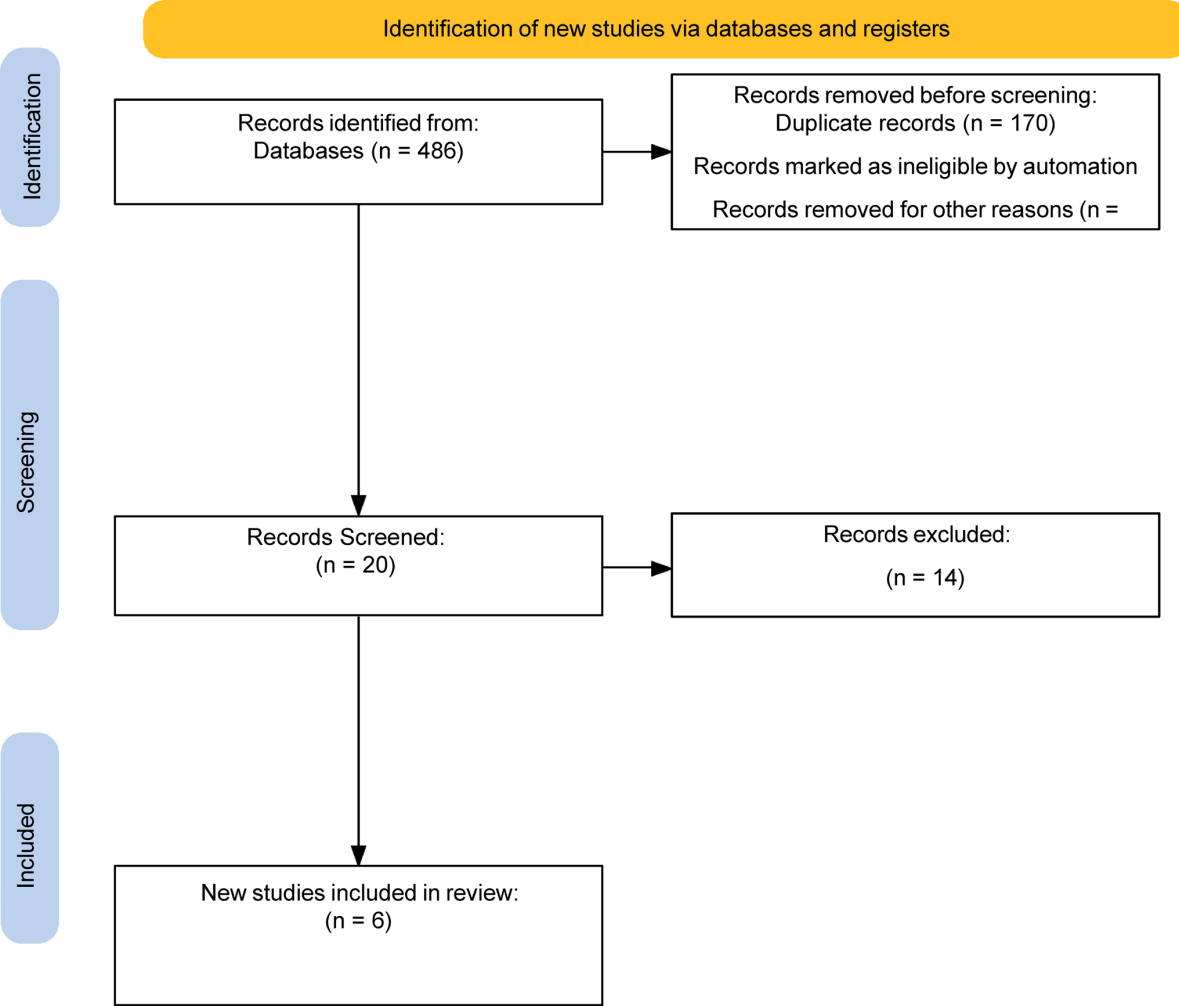
PRISMA flow diagram

Two reviewers (RB, LB) conducted an independent screening of all article titles and abstracts to identify studies potentially relevant to SDD after RAPN. Full-text articles were retrieved for those that appeared to meet inclusion criteria, and eligibility was confirmed based on a thorough review. Any conflicts regarding study inclusion were resolved by consensus with review from a senior author (MS). The study selection process is summarized in Fig. 1, which illustrates a PRISMA flow diagram outlining the identification, screening and inclusion of studies.

For each included study, key data were collected on study design (including country of origin, type of study, and whether it was retrospective or prospective) and basic patient characteristics such as sample size, age, body mass index (BMI), ASA classification, and comorbidity burden as measured by the Charlson Comorbidity Index (CCI). Operative details such tumor complexity, estimated blood loss, surgical approach (retroperitoneal or transperitoneal), ischemia time, and procedure duration were also documented. Studies were examined for information related to SDD protocols, including criteria for discharge.

Readmission rates and visits to the emergency department were also noted. Additional data points included surgical margins, conversion to open surgery, postoperative drain usage, and whether ERAS protocols were implemented, although these were compiled.

The main outcomes evaluated were the rate of successful SDD, complications rates, and readmissions. When available, complications were classified using the Clavien-Dindo system. Additional metrics such as emergency department visits, patient satisfaction and cost data were included where reported.

### Data synthesis

A qualitative synthesis was conducted to summarise the findings from the included studies. Given the variability in the study design, patient populations, and reported outcomes, a formal meta-analysis was not feasible. Instead, results were presented narratively, focusing on key outcomes hospital readmissions, patient satisfaction, and the economic implications of same-day discharge after RAPN. Heterogeneity in study quality and methodology were taken into account during interpretation to ensure a balanced assessment of the evidence.

### Quality assessment

The overall quality of the reviewed studies varied, as most included studies were retrospective in design and therefore subject to inherent limitations such as selection bias and incomplete data reporting. While several studies provided detailed perioperative metrics, such as operative time, ischemia time, and estimated blood loss, others lacked comprehensive outcome data which limited direct comparisons. Sample sizes ranged from small cohorts to larger series, impacting generalizability.

**Fig. 2:**
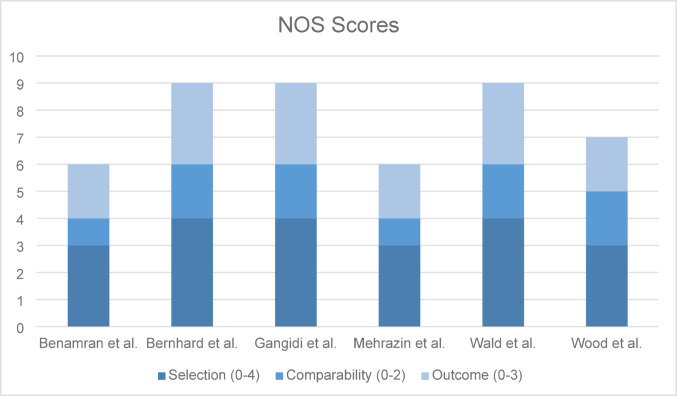
NOS scores for studies included

The Newcastle-Ottawa Scale (NOS) was used to assess study quality. Each study was scored on selection, comparability, and outcome/exposure criteria, the studies were then assigned a score of 0–9, with a score greater or equal to 7 being classified as a high-quality article, while a score of 5–6 was considered moderate quality. The overall scores ranged from moderate to high as shown in Fig. 2. Some demonstrated strong design features such as clear patient selection and detailed outcome reporting. Others, while offering valuable insights were limited by inconsistent follow-up.

## Results

### Study identification

A total of 486 records were identified through database searches. After removal of 466 records prior to screening, 20 studies were assessed for eligibility. Of these, 13 were excluded as they reported outcomes as an inpatient rather than outpatient, and 1 study was excluded due to lack of reporting of outcomes of interest. Ultimately, studies met the inclusion criteria and were incorporated into the final review.

The studies reviewed were mostly conducted in the United States with one based in France, and were all published from 2020 onward, utilizing data collected from 2007 onward. Inclusion and exclusion criteria varied across studies but commonly included assessments of patient comorbidities using the Charlson Comorbidity Index (CCI) and American Society of Anesthesiologists (ASA) classification. Tumor complexity was mostly evaluated using the RENAL nephrectomy score, and tumor size was a standard inclusion metric across studies. Most included tumors were clinical stage T1a (≤ 4 cm), with reported median or average diameters.

ranging from 22 mm to 33 mm [9, 11, 18–21]. Some studies excluded patients if they lived more than 50 km from the hospital, if they lacked home support, if they were on anticoagulation, or if the surgeons deemed an outpatient surgery unfeasible [11, 21]. 

The included studies represented a mix of retrospective, observational and prospective designs. Across the seven studies reviewed, a total of 264 SDD RAPN cases were reported. The number of cases per study ranged from 20 to 82.

Across the included studies, patients had a mean or median age between 55 and 63 years. Reported BMI values ranged from 24 to 29.4 kg/m^2^. Where sex distribution was provided, cohorts were 55–72% male. ASA classification was mostly ASA 2, with smaller proportions of ASA 1 (up to 35%) and ASA 3–4 (up to 67% in some cohorts) depending on the study. Gangidi et al. (2025), noted comparable demographics between SDD and inpatient cohorts but did not provide numeric values. The reported values from Wald et al. (2024), were from 30 SDD patients including both RAPN and robot assisted radical nephrectomy, of whom 21 underwent RAPN. Table 1 below summarizes patient demographics.

Median tumour diameters ranged from 22 mm to 33 mm. Tumor complexity when reported, was typically assessed using the RENAL nephrectomy score, generally falling within the low to moderate range (median scores 5 to 7). Operative times ranged from 60 to 172 min, while ischemia times, when available, ranged from 6 to 24 min. Estimated blood loss was low and ranged between 50 to 120 ml. Reported complication rates were low (3%-10%), with no conversions to open surgery noted in any study. Surgical margins were only recorded in one study and were reported that none of the patients had positive surgical margins [20]. None of the included studies explicitly reported the use of an ERAS protocol. Utilization of postoperative surgical drain was only reported in three studies, all of which reported omitting a surgical drain following surgery [11, 19, 21]. Readmission following emergency department visit was rare and reported only in a few cases with only 1–2 patients being affected [11, 18–21]. Table 2 depicts perioperative outcomes of SDD RAPN.Complications were reported in five of the seven studies, with rates ranging from 4.3% to 10%. The majority were minor (Clavien-Dindo grade I-II), including pain, nausea, anxiety, urinary retention, wound infection and delayed voiding. Only one grade III-IV complication (hematoma) was reported, and no grade V events occurred. Importantly, the clavien -Dindo classification was only used in three of the studies [19–21]. Table 3 summarizes these results. Bernhard et al. (2022) reported an SDD failure rate of 8.5%. 7 patients were kept under observation and discharge the next day with clavien Dindo grade 1 complications. One for Tramadol intolerance with hallucinations, one for anxiety, two for delayed return of normal voiding, and two for uncontrolled pain. No other study in this review reported SDD failure rates

**Table 1:** Patient demographics

Author	Median Age (IQR)	Mean Age	Median BMI (IQR)	Mean BMI	Sex	ASA score
Benamran et al. 2022 [21]	56 (43–66)	NA	27.2 (24.4–29.7)	NA	11 Males (55%) 9 Females (45%)	ASA 1–2 10 (33%) ASA 3–4 20 (67%)
Bernhard et al. 2022 [19]	NA	55 ± 13	NA	Mean 26.2 ± 6.4	59 Males (72%) 23 Females (28%)	Median 2 (1–3)
Gangidi et al. 2025 [9]	NA	NA	NA	NA	NA	NA
Mehrazin et al. 2020 [18]	NA	57.4 ± 12.2	NA	29.38	16 Males (70%) 7 Females (30%)	ASA 2 14 (60.9) ASA 3 9 (39.1%)
Wald et al. 2024 [20]	Median 63 (57–64)	NA	24 (23-26.5)	NA	NA	ASA 1 7 (35%) ASA 2 13 (65%)
Wood et al. 2023 [11]	NA	58.9 ± 9.9	NA	Mean 29.2	46 Males (62%) 28 Females (38%)	ASA 2 45 (60.8%) ASA 3 29 (39.2%)

**Table 2:** Outcomes of SDD RAPN

Author	Platform	Design	Approach	Number of Cases	Tumor Size	Complexity	Operative time	Ischemia time	Estimated Blood Loss	Conversion	Surgical Margins	Drain	ERAS	Readmission	ED
Benamran et al. 2022 [21]	daVinci robot Xi	Observational	13 (65%) Transperitoneal 7 (35%) Retroperitoneal	20	Median 24.5 mm	Median RENAL score 5	Median 60 min	Median 6 min	Median 100 ml	NA	0	0	No	2	NO
Bernhard et al. 2022 [19]	NA	Prospective case series	66 (80%) Transperitoneal, 16 (20%) Retroperitoneal	82	Mean 27 mm	Median RENAL score 7	Mean 111 min	NA	Median 100 ml	NA	NA	0	NO	1.2%	NA
Gangidi et al. 2025 [9]	daVinci Xi	Retrospective Review	79.6% Transperitoneal	44	NA	Low: 45.5% Moderate: 40.9% High: 13.6%	Mean 161.7 min	NA	NA	NA	NA	NA	NO	NA	NA
Mehrazin et al. 2020 [18]	NA	Retrospective	60.9% Transperitoneal	23	Average 22.3 mm	Average RENAL score 6.91	Average 99.5 min	NA	Average 51.1	NA	NA	NA	NO	0	1
Wald et al. 2024 [20]	daVinci Xi	Retrospective	NA	21	22 mm	NA	146 min	NA	100	NA	NA	NA	NO	1	2
Wood et al. 2023 [11]	NA	Retrospective analysis	Transperitoneal	74	Average 33.2	pT1a 50 pT1b 24	Average 172 min	Average 23.7 min	Average 117	NA	NA	0	NO	1	4

## Discussion

### Current state of SDD

SDD following RAPN has emerged as a growing practice in select surgical centers driven by advancements in minimally invasive techniques, and enhanced recovery after surgery (ERAS) protocols, all of which have shortened recovery times, hospital stays, and their associated costs [22]. ERAS protocols have been instrumental in supporting the shift toward SDD after RAPN. These, standardized, evidence-based perioperative care pathways aim to reduce surgical stress, promote early recover, and minimize hospital stays. Key components perioperative counselling, avoidance of prolonged fasting, use of regional or multimodal anaesthesia to limit opioid use, early mobilisation, and prompt return to oral intake [23].

This review demonstrates that the implementation of SDD after RAPN can be safely implemented without compromising short-term perioperative outcomes. Patients discharged on the same day have shown comparable rates of complications, readmission, and emergency department visits when compared to those who remain hospitalized overnight (Table 3).

**Table 3:** Complications by Clavien-Dindo grade (I-V)

Author	Reported Complications	Grade I-II	Grade III-IV	Grade V
Benamran et al. 2022 [21]	2 (10%)	1 (Pain and Anxiety)	1 (Hematoma)	0
Bernhard et al. 2022 [19]	7 (8.5%)	7 (tramadol induced hallucinations, pain, nausea, anxiety, delayed voiding)	0	0
Gangidi et al. 2025 [9]	4 (9.1%)	NA	NA	NA
Mehrazin et al. 2020 [18]	1 (4.3%), Urinary retention	NA	NA	NA
Wald et al. 2024 [20]	1 (4.8%)	1 (Wound infection)	0	0
Wood et al. 2023 [11]	NA	NA	NA	NA

However, the adoption of SDD RAPN remains variable across institutions due to several challenges in implementation. These include logistical considerations such as health care restructuring, and patient access. Clear, evidence-based selection criteria are vital to minimize complications and reduce the risk of failed SDD. Current practices often rely on arbitrary exclusion that don’t account for individual differences. Predictive models may help refine selection and support more personalized decisions [24].

Outpatient RAPN has been slower to gain traction compared to its use in robotic-assisted laparoscopic prostatectomy (RALP) [25]. While SDD is now a more common practice for many RALP procedures, most RAPN patients are still kept overnight for observation [26]. A number of studies have look at overnight stays following RAPN as a more cautious approach, suggesting that full outpatient pathways are still evolving for this type of surgery. Abaza and Shah, 2013, reported that discharge on day 1enha postoperatively is feasible in most patients who had undergone RAPN irrespective of case complexity [27]. The low readmission rates suggest that extended hospital stays may not reduce complications when patients are appropriately selected and meet discharge criteria [27]. Another study by Carbonara et al., found that a single overnight stay after RAPN is feasible and safe [28].

Recent evidence shows that implementing SDD can reduce costs without compromising outcomes. Wood et al. (2023) reported that the introduction of an SDD pathway lowered the mean total cost per patient to $5222 compared with $8425 for inpatient care, reflecting a reduction of over $3000, with no increase in readmissions or emergency department visits [11]. Similarly, Wald, et al. (2024), demonstrated that SDD reduced costs after RAPN by and average of $3091 (18%) and after robotic nephrectomy by $4003 (25%), again without differences in complication rates [20].

### Feasibility of implementing SDD

The studies in this review support the feasibility of SDD following RAPN, with most reporting no differences in complication or readmission rates. Additionally, when applied to carefully selected patients, SDD can potentially reduce overall healthcare costs.

Patient selection is a critical determinant of success for SDD following RAPN, as not all cases are suitable for outpatient management. Careful preoperative assessment is essential to identify individuals who are most likely to benefit from early discharge without compromising safety or outcomes. Factors influencing eligibility include tumor-related characteristics such as size, location, and complexity, all of which can impact the technical difficulty of the procedure and the potential for intraoperative challenges. Equally important are patient-specific factors including overall health status, presence of comorbidities [26], which is commonly assessed using the Charlson Comorbidity Index (CCI) across the studies. Intraoperative variables, including greater estimated blood loss, longer operative times, and prolonged warm ischemia time, have also been linked to longer hospital stays [21]. These considerations highlight the importance of comprehensive approach to patient selection as well as flexibility to adjust planned same day discharge if unexpected intraoperative complications arise.

Another point to consider is the fact that SDD following RAPN might not be feasible outside high-volume centres with experienced urological surgeons and a dedicated outpatient surgery infrastructure [21]. Bernhard et al. (2022), reports that hospital and surgeon volume can play a role in favoring intraoperative outcomes such as low blood loss, and less operating time, ultimately leading to less complications and readmissions [19]. Taking this into account implementing SDD may be challenging for hospitals lacking the necessary surgical volume, expertise and infrastructure.

A recent letter by Crivellaro and Tamborino “*From Crisis to Innovation: The impact of COVID-19 on Outpatient Setting in Robotic Urologic Surgery”* [29] offers important context supporting the feasibility of SDD following RAPN. Using data from the Epic Cosmos dataset, the authors highlight a clear, post-pandemic shift toward outpatient management in robotic urologic procedures, including RAPN and RARP. This transition, accelerated by the need to preserve hospital capacity during COVID-19, reflects growing institutional acceptance of outpatient pathways and aligns with broader adoption of minimally invasive platforms such as the SP robotic system. While the data do not distinguish between surgical techniques or robotic platforms, the large scale of the analysis suggests that outpatient RAPN is becoming more accepted across a range of institutions.

Patient satisfaction and autonomy are also crucial components of successful SDD pathways. Studies have shown that some patients may experience anxiety or discomfort with early discharge, even when medically appropriate [20]. Therefore, it is essential to incorporate shared decision making into the discharge planning process, ensuring patients are well-informed, supported, and comfortable with the plan. This is further supported by findings from a recent study assessing decision regret in patients undergoing SDD after robot-assisted partial prostatectomy, which showed that while the majority of patients reported no regret and would choose the same option again, a notable minority experienced regret, often associated with inadequate pain control, and socioeconomic factors [30]. These insights highlight the importance of individualized discharge planning and proactive postoperative support to optimize patient experience.

### Future directions

Given the advancements in surgical technology and techniques, postoperative care, and patient safety protocols, there is growing interest in SDD. As surgeons become adept with robotic technologies and the evidence supporting SDD increases, it is crucial to evaluate how best to implement SDD practices safely and effectively. Developments in ERAS protocols are essential to supporting SDD. ERAS protocols are designed to streamline recovery processes through evidence-based practices that can significantly decrease the length of stay and enhance postoperative outcomes [31].

Innovation in surgical techniques also stand as a critical factor in enhancing the feasibility of SDD. The use of the retroperitoneal approach might help in increase the adoption of SDD after RAPN. This technique minimizes the risk of intra-abdominal organ injury, provides direct access to the renal hilum, and can result in faster postoperative recovery, reduced complications, and early mobilization [32]. One of the critical advancements is the refinement of robotic surgical systems. The Da Vinci Single Port (SP) system is a notable innovation facilitating less invasive approaches. This system allows for smaller incision and greater manoeuvrability within restricted anatomical spaces. One study indicates that SP RAPN provides comparable outcomes to MP procedures, while also reducing opioid consumptions and enhancing cosmetic results [33, 34]. Abaza et al., compared the outcomes of different urological surgeries using both SP and MP systems, and found that SP surgery is feasible for common urological procedures and allows for a shorter length of stay, and less postoperative pain [35]. Another study also found that SP surgery was associated with a shorter length of stay, but longer warm ischemia times and higher transfusion rates compared to MP [36]. Although cost-specific comparisons between SP and MP RAPN are limited, insights from other robotic procedures, such as robotic-assisted prostatectomy, indicates that SP systems generally incur higher consumable and instrumentation costs. These increased costs are often offset by shorter hospital stays and reduced hospitalization costs, resulting in overall costs that are comparable between SP and MP platforms [37].

Moreover, enhanced remote post-operative recovery care can be potentially leveraged to increase the adaptation of SDD RAPN. As such, telemedicine is emerging as cost-effective and efficient solutions for postoperative follow-up, allowing clinicians to remotely monitor recover while minimizing the need for in-person visits. Along with wearable technologies can also provide better postoperative care by providing real-time data on patients’ postoperative recovery. Devices such as wrist-mounted accelerometers and pedometers and demonstrated feasibility in tracking physical activity, offering valuable insights into early mobilization and functional recovery. These tools may help detect early complications and reduce readmissions [38]. Complementing these tools, AI driven chatbots represent a promising innovation in digital postoperative care. In urology, chatbots can guide patients through their recovery by delivering personalized reminders, medication schedules, wound care instruction, and basic symptom triage. However, barriers such as limited access to high-speed internet, digital illiteracy and language or socioeconomic disparities must be addressed to ensure equitable access [39, 40].

### Limitations of this study and available literature

Despite growing interest in SDD following RAPN, the available literature remains limited in both scope and quality. The studies included in this review are mostly retrospective, center-specific, which restricts the generalizability of the findings. Additionally, the studies reflect outcomes from specialized, high-performing surgical units, often led by experienced surgeons. As a result, the data may not reflect outcomes achievable in more typical clinical environments, where surgical volume and resources vary significantly.

Another challenge is the lack of uniform reporting standards across studies. Definitions of key outcomes such as discharge failure, postoperative complications, and follow-up protocols differ widely, making meaningful comparisons difficult. The absence of standardized ERAS protocols also complicates interpretation as it is unclear which specific perioperative strategies contribute to successful SDD. Future studies should strive to establish standardized reporting outcomes to aid in data aggregation and multi-site studies.

The literature also offers limited insight into how institutional infrastructure, staffing, or perioperative logistics influence the success of SDD. Without this context, it is unclear which findings are tied to replicable protocols, and which are dependent on specific conditions that may not be easily duplicated.

## Conclusions

SDD following RAPN appears safe and feasible in carefully selected patients, with outcomes comparable to inpatient care. However, the evidence remains preliminary due to studies quality and variability. Broader adoption will require standardized protocols, improved patient selection tools, and a prospective, multicenter registry.

## Data Availability

Not applicable.

## Declarations

### Purpose of review

To discuss the current landscape of SDD for RAPN, review the current literature on the topic, and propose future research on SDD.
